# Clinical Lectures on Pulmonary Consumption

**Published:** 1854-08

**Authors:** 


ART IV.—*Clinical Lectures on Pulmonary Consumption.* By THEOPHILUS THOMPSON, M. D., F. R. S., Fellow of the Royal College of Physicians, London; Physician to the Hospital for Consumption and Diseases of the Chest; Author of the Annals of Influenza, etc., etc. Philadelphia: Lindsay & Blakiston. 1854.

It is fortunate for humanity that there are hopeful minds in the profession; minds not readily abandoning all effort in even the most difficult paths of therapeutical research. The appearance of Bennett on Pulmonary Tuberculosis in Scotland, (soon to be republished in this country) afforded us occasion, last winter, to speak in hopeful phrase of the final result of the research into the causation and cure of tubercle. Dr. Bennett's was in great measure a theoretical work, treating of cause more fully than of cure, investigating the hidden processes of nutrition rather than the more potent manifestations of its results. One of the ablest and most valuable of monographs, it still left a blank space between theory and practice in the history of the disease; a space which the present "Clinical Lectures" admirably occupy.

Dr. Thompson occupies the position of physician to the hospital for consumptives, at Brompton, near London; a humane institution, where great good is being accomplished; and which affords the finest opportunities for the study of tubercle. From the records of this hospital, and from the cases it contains, Dr. Thompson has been able to furnish a most instructive succession of cliniques.

In the introduction, a change in the nomenclature of sounds is proposed, with a view to the simplification of their study, and to removing from some of them a certain ambiguity of meaning.

To this end he divides the sounds into five classes, viz: 1, Bubbling; 2, Clicking; 3, Crackling; 4, Crepitation; and 5, Vibration. These names are substituted for their numerous synonyms as commonly used. The word rhoncus is rejected from the clicking, crackling, and crepitant sounds; and is only used in describiug the bubbling, and vibrating sounds. In the bubbling sounds we have the *bubbling rhoncus*, (mucous rhoncus) of bronchitis; the *small bubbling rhoncus*, (sub-crepitant rhoncus) of capillary bronchitis; and *gurgling*, (cavernous rhoncus) of excavation. The *clicking* sound is confined to softening tubercle, the *crackling* to unsoftened tubercle, and *crepitation* to pneumonia; while *vibration* includes the sonorous and sibilant rhonchi of bronchial asthma, &c.

This division and nomenclature of the more common and important sounds, seems, to us, simple, and preferable to that mixed nomenclature, which confuses the sound and its only occasional cause in a single word of doubtful meaning.

Dr. Thompson, like other recent authors, gives in a full adhesion to the value of cod liver oil in tubercle. He esteems it the best and most reliable remedy, and has found no substitute for it among the vegetable oils, with the exception of cocoa-nut oil, with which he has obtained some very encouraging results. The cheapness of this oil, its pleasant flavor, and the large sources for its supply, render it a most eligible substitute, if it should be found to exert an equal influence on the assimilative function. We have not expected to find any such article out of the animal kingdom, but having, in our own experience, met with great difficulty in persuading patients to take the necessary quantity of cod liver oil, we have found that fat meats, and a large allowance of butter and other greasy food, were very efficient nutritives; and we think we have derived from them the same benefit as from the oil. We have not as yet met with any trouble from indigestion. In fact, the function of the stomach has so little to do with the digestion of fatty food, that we see no reason why it should excite dyspepsia.

We do not recollect to have met, elsewhere, with any mention of what the author calls the *gingival margin*, as a diagnostic sign of tubercle. This appearance is, in the words of the author, "a mark on the reflected edge of the gums, usually deeper in color than the adjoining surface, and producing a festooned appearance, by the accuracy with which it corresponds with the curve of the gingival border; this mark is in some patients a mere streak; in others, a margin, sometimes more than a line in breadth. In the most decided cases, this margin is of a vermillion tint, inclining to lake. It is worthy of remark that * * * * a colored line also strongly marks the place where the mucous membrane of the lower lips is reflected on the gums. As a general rule, the line is most distinct around the incisor teeth, but it is frequently apparent also round the molars."

Of twenty-six men, twenty had the margin. All these were phthisical. Of the six who had not the margin, one, a boy of twelve years, was tubercular; the others had other diseases. Dr. Thompson supposes that it does not as often exist in children. It is also less common among patients in the better classes, than among hospital, or poor patients.

Of twenty-one phthisical women, eight were without the margin. No reason for this difference in the sexes has been discovered. Dr. T. reckons it among the early signs of tubercle, and regards its absence as a favorable sign; though, as will be seen, he does not consider it a diagnostic sign.

On the whole, we look upon this as a valuable contribution to the literature of tubercle. Dr. Meigs recently stated, that, since the introduction of cod liver oil, the mortality from phthisis has materially diminished in the city of Phlladelphia. He thinks that one-eighth of consumptive cases are now cured. We think that the observation of intelligent practitioners will confirm this statement, and that consumption, so far from being the "*opprobrium medicinæ*," is in truth one of the greatest glories of legitimate medicine.

Very much patient research is, in this, bringing forth a good reward; and it is not too much to hope, that the hygienic and curative means now at the disposal of the physician, may still further reduce the mortality from this source. H.

